# Trajectories of cognitive processing speed and physical disability over 11 years following initiation of a first multiple sclerosis disease-modulating therapy

**DOI:** 10.1136/jnnp-2023-331784

**Published:** 2023-08-09

**Authors:** Elisa Longinetti, Simon Englund, Joachim Burman, Katharina Fink, Anna Fogdell-Hahn, Martin Gunnarsson, Jan Hillert, Annette Magdalene Langer-Gould, Jan Lycke, Petra Nilsson, Jonatan Salzer, Anders Svenningsson, Johan Mellergård, Tomas Olsson, Fredrik Piehl, Thomas Frisell

**Affiliations:** 1 Clinical Epidemiology Division, Department of Medicine Solna, Karolinska Institutet, Stockholm, Sweden; 2 Department of Clinical Neuroscience, Karolinska Institutet, Stockholm, Sweden; 3 Department of Medical Sciences, Uppsala University, Uppsala, Sweden; 4 Department of Neurology, Örebro University, Orebro, Sweden; 5 Clinical and Translational Neuroscience, Kaiser Permanente Southern California, Pasadena, California, USA; 6 Department of Clinical Neuroscience, University of Gothenburg, Goteborg, Sweden; 7 Department of Clinical Sciences, Division of Neurology, Lund University, Lund, Sweden; 8 Department of Clinical Sciences, Neurosciences, Umeå University, Umeå, Sweden; 9 Department of Clinical Sciences, Danderyd Hospital, Karolinska Institutet, Stockholm, Sweden; 10 Department of Neurology, Linköping University, Linkoping, Östergötland, Sweden; 11 Department of Biomedical and Clinical Sciences, Linköping University, Linköping, Sweden

**Keywords:** epidemiology, multiple sclerosis, cognition

## Abstract

**Background:**

We analysed the COMparison Between All immunoTherapies for Multiple Sclerosis (NCT03193866), a Swedish nationwide observational study in relapsing-remitting multiple sclerosis (RRMS), to identify trajectories of processing speed and physical disability after disease-modulating therapy (DMT) start.

**Methods:**

Using a group-modelling approach, we assessed trajectories of processing speed with oral Symbol Digit Modalities Test (SDMT) and physical disability with Expanded Disability Status Scale, from first DMT start among 1645 patients with RRMS followed during 2011–2022. We investigated predictors of trajectories using group membership as a multinomial outcome and calculated conditional probabilities linking membership across the trajectories.

**Results:**

We identified 5 stable trajectories of processing speed: low SDMT scores (mean starting values=29.9; 5.4% of population), low/medium (44.3; 25.3%), medium (52.6; 37.9%), medium/high (63.1; 25.8%) and high (72.4; 5.6%). We identified 3 physical disability trajectories: no disability/stable (0.8; 26.8%), minimal disability/stable (1.6; 58.1%) and moderate disability (3.2; 15.1%), which increased to severe disability. Older patients starting interferons were more likely than younger patients starting rituximab to be on low processing speed trajectories. Older patients starting teriflunomide, with more than one comorbidity, and a history of pain treatment were more likely to belong to the moderate/severe physical disability trajectory, relative to the no disability one. There was a strong association between processing speed and physical disability trajectories.

**Conclusions:**

In this cohort of actively treated RRMS, patients’ processing speed remained stable over the years following DMT start, whereas patients with moderate physical disability deteriorated in physical function. Nevertheless, there was a strong link between processing speed and disability after DMT start.

**WHAT IS ALREADY KNOWN ON THIS TOPIC**

- Despite cognitive impairment being one of the most common and debilitating symptoms of multiple sclerosis, it is unclear how cognitive impairment and physical disability of relapsing-remitting multiple sclerosis (RRMS) patients change over longer periods of time following first disease-modulating therapy (DMT) initiation in a contemporary therapeutic setting.

**WHAT THIS STUDY ADDS**

- Unlike physical disability, processing speed, if affected already at DMT initiation, remained stable in this cohort of 1645 actively treated and monitored patients with RRMS followed during 2011–2022. This difference aside, there was a strong link between processing speed and disability after DMT start.

**HOW THIS STUDY MIGHT AFFECT RESEARCH, PRACTICE OR POLICY**

- We observed that RRMS patients’ processing speed remained stable over an average of 7 years following DMT initiation, but we observed that older age and severe disease were associated with cognitive impairment at baseline, in turn highlighting the importance of early intervention to maintain cognitive functions in RRMS.

## Introduction

A common and debilitating symptom of multiple sclerosis (MS) is cognitive impairment, affecting 40%–70% of people living with MS.^1^ Cognitive impairment in MS^2^ is reported to be related to the accumulation of disability^3–5^ and is associated with increased grey matter atrophy of hippocampus^6^ and thalamus,^7^ reflecting their relevance for memory and attention.

One of the most commonly affected cognitive domains is processing speed,^8^ the ability to quickly and accurately process information. Essential for everyday tasks such as reading, writing and problem-solving, processing speed is significantly impaired in individuals with MS,^9 10^ leading to substantial impact on their ability to work and overall quality of life.^11^ Despite this, high-quality data from well-powered studies on the evolution of processing speed in patients with MS, its predictors and association with physical disability worsening are still scarce.^12 13^

A widely used measure of cognitive function in individuals with MS is the Symbol Digit Modalities Test (SDMT), which has been found to be sensitive to cognitive impairment in MS as a measure of processing speed.^10 14^ By reporting decreasing scores on the SDMT, where a lower score reflects a higher impact on cognitive processing, several studies indicate that the processing speed of patients with MS worsens over time.^12 13 15–17^ The treatment may impact on this risk, or even be associated with improvement, as suggested by observational data for individuals initiating natalizumab.^17 18^ However, it is unclear if this effect can be in part explained by the practice effect of performing repeated tests.^19^

In addition to lower baseline SDMT scores,^15^ other suggested predictors of cognitive impairment^12 20–22^ and decline in SDMT scores^13^ in MS are increasing age,^13 15 21^ disease duration,^15 20^ physical disability,^12 13 20 23^ brain volume loss,^12 13^ lesion load,^13^ vocabulary,^20^ lower cognitive reserve (measured by years of education),^14^ depression^24^ and anxiety.^22^

However, larger population-based studies with long follow-up leveraging high-quality data are needed to better predict the long-term course of cognitive impairment in MS. An important additional aspect is also to explore evolution of cognitive functions in contemporary MS cohorts exposed to disease-modulating therapy (DMT). To fill these knowledge gaps, we followed a cohort of 1645 relapsing-remitting MS (RRMS) patients for up to 11 years from the start of their first DMT, linking data from the Swedish nationwide observational study in RRMS, COMparison Between All immunoTherapies for Multiple Sclerosis (COMBAT-MS), to several Swedish national registers. We aimed to (1) identify trajectories of processing speed and physical disability and their connections and (2) describe patient characteristics associated with trajectory groups.

## Methods

In a population-based cohort study of patients with RRMS, we assessed trajectories of processing speed and disability linking COMBAT-MS data to the Swedish MS Registry and national healthcare and census registers. To be part of COMBAT-MS, patients had to sign an informed consent covering information regarding data handling, secrecy, and safety.

### Study design

The COMBAT-MS (NCT03193866) is, to the best of our knowledge, the largest population-based observational study in RRMS. Out of an eligible study population of 3800 (~50% of the nationwide prevalence), The COMBAT-MS cohort enrolled about 3500 patients with MS (92%) who started a new DMT between 2011 and 2018 at the MS clinics of any of the university hospitals of Sweden. Annual prospective data collection was conducted from 2017 to 2022, and recorded through the Swedish MS Registry,^25^ an integrated web-based part of Swedish MS care collecting accurate and complete^26^ healthcare data for all patients with MS in Sweden since 2001. Prior to 2017, chart validation of data entered into the Swedish MS Registry was done retrospectively.^26^

### Study population

Our study population included all patients with RRMS, 18 years or older, enrolled in the COMBAT-MS study, starting a first DMT with dimethyl fumarate, fingolimod, glatiramer acetate, interferons (interferon beta-1a, peginterferon beta-1a and interferon beta-1b), natalizumab, rituximab or teriflunomide, consented to be included in the Swedish MS Registry, residing in Sweden from 5 years prior to MS diagnosis or longer, with at least three oral SDMT scores recorded after first DMT start (N=1645). Through the unique personal identification numbers assigned to all Swedish residents, we cross-linked our study population to the Swedish Migration^27^ and Causes of Death Register,^28^ to follow the cohort from first DMT start until emigration from Sweden, death, withdrawal from the Swedish MS Registry or end of follow-up (12 May 2022), whichever came first. Participants were followed regardless of DMT switch or discontinuation.

### Processing speed and disability ascertainment

Processing speed and physical disability were defined via oral SDMT and Expanded Disability Status Scale (EDSS) scores, respectively, recorded initially as part of the annual neurological assessment in routine care and, from 2017, as part of COMBAT-MS. To attenuate the test practice effect,^19^ annual switching of SDMT versions were coordinated centrally from 2017 onward. We excluded from the analyses written SDMT scores, due to the non-interchangeable mode of SDMT administration.^29^

### Potential predictors of processing speed and physical disability trajectories

To extract data on potential predictors of processing speed and physical disability trajectories at DMT start, we cross-linked the Swedish MS registry to the following demographic and health registries: population, patient, prescribed drug, and data from the Swedish social insurance agency and longitudinal database for insurance and labour market studies. We collected information on age, sex, country of birth, region of residence, educational level, comorbid conditions (including the Charlson Comorbidity Index,^30^ depression, anxiety disorders and other psychiatric comorbidities), prescribed treatment dispensation, DMT, MS duration, recent relapse, recent cerebral lesions, baseline scores of SDMT, EDSS, Fatigue Scale for Motor and Cognitive function (FSMC) total, the physical and psychological domains of the MS Impact Scale (MSIS-29) and EuroQol Visual Analogue Scale (EQ-VAS), days on sick leave and disability pension (definitions: table 1 and online supplemental eTable 1).

10.1136/jnnp-2023-331784.supp1 Supplementary data

**Table 1:** Characteristics of study participants at first DMT start according to processing speed trajectories (N=1645)

	Processing speed trajectories according to SDMT starting values, N (%)				
	Low	Low/medium	Medium	Medium/high	High
N (%)	89 (5.4)	416 (25.3)	630 (38.3)	419 (25.5)	91 (5.5)
Age at DMT start (years)					
18–34	23 (25.8)	146 (35.1)	287 (45.6)	253 (60.4)	66 (72.5)
>34	66 (74.2)	270 (64.9)	343 (54.4)	166 (39.6)	25 (27.5)
Female	54 (60.7)	266 (63.9)	453 (71.9)	312 (74.5)	63 (69.2)
Born in Sweden	71 (79.8)	349 (83.9)	555 (88.1)	386 (92.1)	80 (87.9)
Education over 12 years	26 (29.2)	166 (40.0)	334 (53.2)	269 (64.4)	68 (74.7)
Comorbidity≥1*	15 (16.9)	51 (12.3)	62 (9.8)	31 (7.4)	7 (7.7)
Depression diagnosis†	9 (10.1)	28 (6.7)	36 (5.7)	14 (3.3)	1 (1.1)
Anxiety diagnosis†	9 (10.1)	47 (11.3)	58 (9.2)	23 (5.5)	5 (5.5)
Other psychiatric comorbidities†‡	15 (16.9)	40 (9.6)	40 (6.3)	16 (3.8)	2 (2.2)
Antidepressants treatment§	20 (22.5)	69 (16.6)	71 (11.3)	39 (9.3)	8 (8.8)
Anxiolytics treatment§	7 (7.9)	22 (5.3)	24 (3.8)	10 (2.4)	4 (4.4)
Symptomatic fatigue treatment§	1 (1.1)	13 (3.1)	9 (1.4)	2 (0.5)	1 (1.1)
Sleeping aids treatment§	16 (18.0)	61 (14.7)	83 (13.2)	35 (8.4)	10 (11.0)
Pain treatment§	35 (39.3)	138 (33.2)	207 (32.9)	123 (29.4)	22 (24.2)
DMT					
Dimethyl fumarate	15 (16.9)	85 (20.4)	113 (17.9)	79 (18.9)	19 (20.9)
Fingolimod	1 (1.1)	18 (4.3)	29 (4.6)	14 (3.3)	6 (6.6)
Glatiramer acetate	1 (1.1)	21 (5.0)	24 (3.8)	12 (2.9)	4 (4.4)
Interferons¶	31 (34.8)	117 (28.1)	189 (30.0)	121 (28.9)	32 (35.2)
Natalizumab	8 (9.0)	56 (13.5)	84 (13.3)	78 (18.6)	18 (19.8)
Rituximab	31 (34.8)	102 (24.5)	172 (27.3)	105 (25.1)	12 (13.2)
Teriflunomide	2 (2.2)	17 (4.1)	19 (3.0)	10 (2.4)	0 (0.0)
MS duration (years)					
0–5	84 (94.4)	385 (92.8)	584 (93.1)	397 (95.0)	88 (96.7)
>5	5 (5.6)	30 (7.2)	43 (6.9)	21 (5.0)	3 (3.3)
Relapse previous year	52 (58.4)	265 (63.7)	422 (67.0)	295 (70.4)	65 (71.4)
New cerebral lesion previous year	45 (56.3)	163 (43.6)	279 (50.5)	179 (48.4)	43 (51.8)
SDMT Score, mean (SD)	29.4 (7.2)	43.8 (6.8)	52.1 (6.2)	62.5 (8.4)	72.0 (10.4)
EDSS Score, mean (SD)	2.7 (1.2)	2.1 (1.3)	1.7 (1.2)	1.4 (1.1)	1.4 (1.0)
FSMC total Score, mean (SD)	64.7 (25.3)	52.0 (20.8)	44.4 (20.5)	41.2 (19.5)	39.0 (14.9)
MSIS-29 Physical Score, mean (SD)	41.5 (24.8)	26.5 (21.5)	16.6 (18.8)	13.2 (15.9)	11.3 (15.9)
MSIS-29 Psychological Score, mean (SD)	51.9 (25.2)	40.3 (26.2)	34.5 (23.8)	29.9 (23.1)	26.4 (20.6)
EQ-5D VAS Score, mean (SD), mean (SD)	53.0 (26.9)	63.8 (21.6)	71.6 (20.6)	75.2 (19.2)	79.1 (14.7)
Sick leave previous year, mean (SD), days**	58.5 (102.1)	28.9 (57.4)	20.7 (53.2)	11.1 (31.9)	9.3 (28.9)
Disability pension previous year, mean (SD), days**	49.7 (119.4)	18.5 (74.6)	4.4 (33.7)	2.1 (24.6)	0.0 (0.0)

*Diagnosed within 5 years prior to DMT start according to the Charlson Comorbidity Index.

†Diagnosed within 5 years prior to DMT start.

‡All mental and behavioural disorders except depression and anxiety disorders.

§Dispensed prescribed drugs within 1 year prior to DMT start.

¶Interferon beta-1a, peginterferon beta-1a and interferon beta-1-b.

**Restricted to patients 18–64 years old.

DMT, disease-modulating therapy; EDSS, Expanded Disability Status Scale; EQ-VAS, EuroQol Visual Analogue Scale; FSMC, Fatigue Scale for Motor and Cognitive function; MS, multiple sclerosis; MSIS-29, MS Impact Scale; SDMT, Symbol Digit Modalities Test.

### Statistical analysis

We identified distinctive clusters of individual SDMT and EDSS trajectories using group-based trajectory modelling, an application of latent growth mixture models.

In censored normal models, we linked time since first DMT start and SDMT and EDSS scores through a polynomial relationship. We conducted model selection in two steps using the Bayesian information criteria.^31^ First, we tested the optimal number of trajectory groups by using a quadratic form for all trajectory groups and the recommended 5% minimum group size requirement.^32^ Then, we identified the appropriate order of the polynomial function used to model each group’s trajectory by specifying the shape of each group up to a cubic function. For processing speed, the model with five trajectories and a linear, and four cubic functions of time since DMT start showed the best fit to the data, whereas for physical disability, it was the model with three trajectories, a cubic, a linear and a quadratic function. We assigned participants into the trajectory to which their average posterior probability (AvePP) of assignment was greatest and visualised trajectories as the maximum-likelihood estimates plotted against time since first DMT start for intuitive interpretation. The AvePP was 0.96, 0.92, 0.91, 0.94, 0.96 for processing speed trajectories and 0.96, 0.97, 0.96 for disability trajectories, well above the AvePP≥0.70 recommended model adequacy criteria.^31^

To understand how individual characteristics may influence the developmental course of SDMT and EDSS trajectories, we investigated potential determinants of trajectories (as listed above) by using group membership assignments as a multinomial outcome and derived ORs and the corresponding 95% CIs from multinomial logistic regression models. Each potential predictor was first analysed separately and then progressively inserted into the full model.

We used joint trajectory models^33^ to analyse connections between the developmental course of SDMT and EDSS as two distinct but related outcomes and reported conditional probabilities linking membership across the trajectory groups of the two respective scales.

### Missing data

Prior to the identification of SDMT and EDSS trajectories via group-based trajectory modelling, we addressed missing baseline covariate values with multiple imputation using chained equations. Each missing variable was imputed as a function of all other baseline variables used in the analysis (plus their transformations), and the predicted trajectory group membership probabilities. Predictive mean matching was used for quantitative variables and logistic regression for categorical variables, with models defined using fully conditional specification, 20 imputations and 10 burn-in iterations. CIs around group contrasts were constructed by pooling effect estimates and variance using Rubin’s rules.^34^ By imputing missing baseline covariate values with multiple imputation using chained equations, we assume a missing data mechanism that is missing at random, enabling imputation based on observed variables and the imputation model includes all relevant potential predictors for accurate imputed values. Consequently, accurate imputed baseline values facilitate the identification of SDMT and EDSS trajectories via group-based trajectory modelling, accounting for associations with other patient characteristics.

### Sensitivity analyses

To account for trajectory misclassification, we investigated potential determinants of trajectories after excluding participants whose trajectory assignment probability was below 0.80.

All analyses were made with Stata V.16.1 and SAS V.9.4.

## Results

Between 2011 and 2022, 1645 study participants were followed for an average of 7.1 years (2.2 SD) from DMT start, with mean numbers of SDMT and EDSS scores collected being 7.3 (3.8 SD, 73.3% in the context of the COMBAT-MS study) and 7.3 (3.9 SD, 63.8% in the context of the COMBAT-MS study), respectively (online supplemental eTable 2: number of patients with SDMT and EDSS scores at each time point). The average time between MS diagnosis and first DMT was 0.96 years (3.24 SD). During follow-up, 3 individuals died, and 10 emigrated from Sweden.

### Processing speed and physical disability trajectories

**Figure 1:**
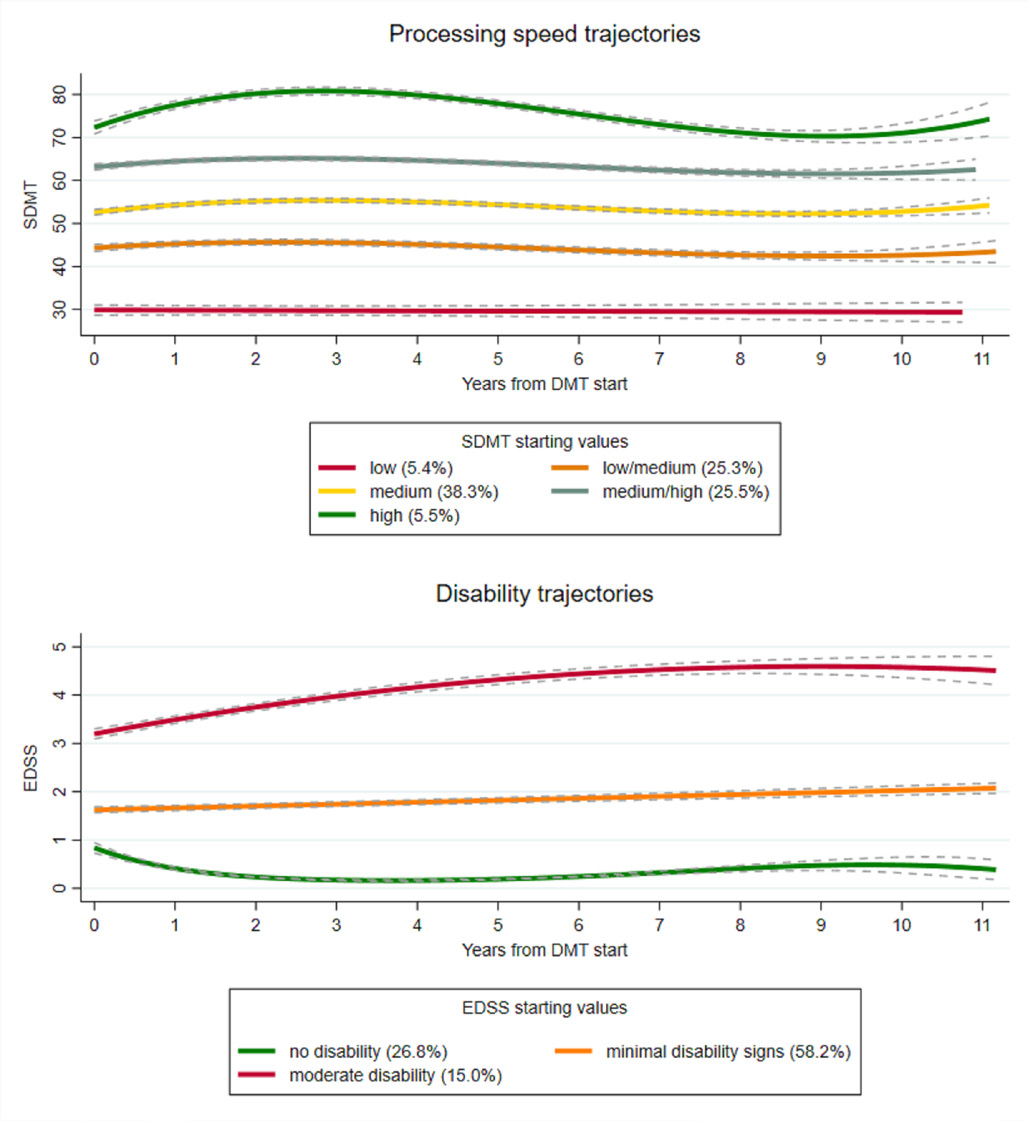
Processing speed (Symbol Digit Modalities Test (SDMT)), disability (Expanded Disability Status Scale (EDSS)) trajectories and corresponding 95% CIs, over 11 years following first disease-modulating therapy (DMT) initiation.

The group-based trajectory modelling algorithm identified five processing speed trajectories and three physical disability trajectories. Figure 1 shows the estimated mean SDMT scores in the 5 processing speed trajectories at each year following DMT start: 5.4% of individuals maintained low SDMT scores (mean starting values (MSV)=29.9) over 11 years, 25.3% maintained low/medium SDTM scores (MSV=44.3), 37.9% maintained medium SDMT scores (MSV=52.6), 25.8% maintained medium/high SDMT scores (MSV=63.1) and 5.6% maintained high SDMT scores (MSV=72.4). Although the low/medium, medium, medium/high and high processing speed trajectories slightly increased the first 5 years following DMT start, they decreased in the subsequent years. Thus, despite an initial minimally clinically meaningful difference (defined as>8 SDMT points^35^) in the high processing trajectory between 2.3 and 4.6 years after DMT start, no minimally clinically meaningful differences remained at the end of follow-up.

Figure 1 also shows the estimated mean EDSS scores in the 3 disability trajectories at each year following DMT start: 26.8% of individuals maintained no disability (MSV=0.8), 58.1% maintained minimal disability (MSV=1.6) and 15.1% increased from moderate disability (MSV=3.2) to severe disability (mean values at the end of follow-up=4.8). From 4 years after DMT start, the observed increase of the moderate disability trajectory to severe disability constituted a clinically relevant change (defined as >1 EDSS point^36^). The observed slight decrease of the no disability trajectory during the first 4 years following DMT start, and the slight increase over time of the minimal disability signs trajectory, did not constitute a clinically relevant change.

### Predictors of processing speed and disability trajectories

Sociodemographic, clinical and MS-related characteristics of the study participants at DMT start, stratified by the identified processing speed trajectories, are shown in table 1, with adjusted comparisons in table 2.

**Table 2:** ORs of belonging to processing speed trajectories (compared with the low processing speed one) in a multivariable model including region of residence and baseline SDMT in addition to all potential predictors listed, N=1645 patients with RRMS on first DMT

	Processing speed trajectories, SDMT starting values			
	Low/medium	Medium	Medium/high	High
	OR (95% CI)	OR (95% CI)	OR (95% CI)	OR (95% CI)
Age, years				
18–34	Ref.	Ref.	Ref.	Ref.
>34	0.57 (0.29 to 1.11)	**0.39 (0.19 to 0.80)**	**0.24 (0.11 to 0.50)**	**0.17 (0.07 to 0.40)**
Female versus male	1.45 (0.47 to 4.51)	2.05 (0.61 to 6.94)	2.09 (0.56 to 7.77)	1.30 (0.31 to 5.34)
Born in Sweden versus born outside Sweden	0.90 (0.20 to 4.06)	1.12 (0.22 to 5.70)	1.29 (0.22 to 7.42)	0.72 (0.09 to 5.57)
Years of education>12 vs ≤12	1.32 (0.38 to 4.62)	1.68 (0.43 to 6.57)	1.79 (0.41 to 7.80)	2.52 (0.51 to 12.43)
Comorbidity≥1* versus none	0.48 (0.08 to 2.88)	0.33 (0.05 to 2.24)	0.17 (0.02 to 1.33)	0.13 (0.01 to 1.38)
History of depression†, yes versus no	0.28 (0.02 to 4.27)	0.29 (0.02 to 5.22)	0.12 (0.00 to 2.90)	n/a
History of anxiety†, yes versus no	2.78 (0.33 to 23.70)	2.59 (0.27 to 24.73)	2.44 (0.21 to 28.40)	6.17 (0.32 to 119.94)
History of other psychiatric comorbidities†‡, yes versus no	0.42 (0.05 to 3.29)	0.31 (0.04 to 2.69)	0.18 (0.02 to 1.98)	n/a
History of antidepressants treatment§, yes versus no	0.87 (0.14 to 5.45)	0.62 (0.09 to 4.42)	0.45 (0.06 to 3.66)	0.53 (0.05 to 5.09)
History of anxiolytics treatment§, yes versus no	0.47 (0.03 to 6.55)	0.34 (0.02 to 6.78)	0.50 (0.02 to 15.36)	n/a
History of symptomatic fatigue treatment§, yes versus no	n/a	n/a	n/a	n/a
History of sleeping aids treatment§, yes versus no	2.11 (0.39 to 11.38)	2.50 (0.43 to 14.62)	2.11 (0.30 to 14.73)	4.32 (0.52 to 35.98)
History of pain treatment§, yes versus no	0.61 (0.18 to 2.10)	0.56 (0.15 to 2.12)	0.47 (0.11 to 2.02)	0.28 (0.05 to 1.49)
DMT				
Rituximab	Ref.	Ref.	Ref.	Ref.
Dimethyl fumarate	0.42 (0.08 to 2.06)	0.33 (0.06 to 1.77)	0.32 (0.05 to 1.85)	n/a
Fingolimod	n/a	n/a	n/a	n/a
Glatiramer acetate	n/a	n/a	n/a	n/a
Interferons¶	**0.21 (0.05 to 0.90)**	**0.16 (0.03 to 0.80)**	**0.14 (0.02 to 0.90)**	n/a
Natalizumab	7.75 (0.68 to 88.18)	**15.69 (1.28 to 192.03)**	**59.63 (4.11 to 864.23)**	n/a
Teriflunomide	n/a	n/a	n/a	n/a
MS duration>5 years vs ≤5 years	0.96 (0.25 to 3.74)	0.97 (0.23 to 4.00)	0.79 (0.17 to 3.69)	n/a
Any relapse versus none in the previous year	1.67 (0.57 to 4.90)	1.94 (0.60 to 6.29)	2.36 (0.63 to 8.88)	2.38 (0.55 to 10.37)
Any new cerebral lesions versus none in the previous year	0.44 (0.14 to 1.43)	0.59 (0.16 to 2.18)	0.53 (0.13 to 2.21)	0.58 (0.12 to 2.90)
EDSS Score	0.83 (0.49 to 1.41)	0.75 (0.43 to 1.32)	0.76 (0.42 to 1.39)	0.92 (0.45 to 1.87)
FSMC Score	1.05 (0.96 to 1.15)	1.03 (0.94 to 1.14)	1.04 (0.94 to 1.16)	1.01 (0.91 to 1.12)
MSIS-29 Physical Score	0.96 (0.90 to 1.03)	0.95 (0.89 to 1.02)	0.95 (0.88 to 1.02)	0.96 (0.88 to 1.03)
MSIS-29 Psychological Score	0.99 (0.95 to 1.03)	1.00 (0.95 to 1.04)	0.99 (0.94 to 1.03)	0.98 (0.93 to 1.04)
EQ-5D VAS Score	1.01 (0.96 to 1.07)	1.02 (0.96 to 1.07)	1.02 (0.96 to 1.07)	1.01 (0.94 to 1.08)
Sick leave previous year**, days	1.00 (0.99 to 1.01)	1.00 (0.99 to 1.01)	1.00 (0.99 to 1.01)	1.00 (0.98 to 1.02)
Disability pension previous year, days**	1.00 (0.99 to 1.01)	1.00 (0.99 to 1.00)	1.00 (0.99 to 1.01)	0.92 (0.05 to 16.36)

Bold values denote statistical significance at the p < 0.05 level.

*Diagnosed within 5 years prior to DMT start according to the Charlson Comorbidity Index.

†Diagnosed within 5 years prior to DMT start.

‡All mental and behavioural disorders except depression and anxiety disorders.

§Dispensed prescribed drugs within 1 year prior to DMT start.

¶Interferon beta-1a, peginterferon beta-1a and interferon beta-1b.

**Restricted to patients 18–64 years old.

DMT, disease-modulating therapy; EDSS, Expanded Disability Status Scale; EQ-VAS, EuroQol Visual Analogue Scale; FSMC, Fatigue Scale for Motor and Cognitive function; MS, multiple sclerosis; MSIS-29, MS Impact Scale; n/a, not applicable; RRMS, relapsing-remitting MS; SDMT, Symbol Digit Modalities Test.

Before imputation, information at DMT start was missing for <1% for all potential predictors of processing speed and disability trajectories, except new cerebral lesions, SDMT, EDSS, FSMC total, MSIS-29 and EQ-5D VAS scores (11%–83%, online supplemental eTable 3).

After mutual adjustment for all potential predictors tested, study participants older than 34 years at DMT start were significantly less likely to belong to the medium, medium/high and high processing speed trajectories, relative to the low processing speed one, than participants aged 18–34 years, as were participants starting interferons, than participants starting rituximab (table 2). Being female, born in Sweden, and having>12 years of education were positively associated with medium and higher processing speed trajectories (table 1) but the association was no longer significant after adding MS disease-related scores into the model (table 2).

Study participants older than 34 years at DMT start were also more likely to belong to the minimal disability signs and moderate disability trajectories, relative to the no disability one, than participants aged 18–29 years (table 3).

**Table 3:** ORs of belonging to disability trajectories (compared with the no disability one) in a multivariable model including region of residence and baseline EDSS in addition to all potential predictors listed, N=1645 patients with RRMS on first DMT

	Disability trajectories, EDSS starting values	
	Minimal disability signs	Moderate disability
	OR (95% CI)	OR (95% CI)
Age, years		
18–34	Ref.	Ref.
>34	**1.19 (1.03 to 1.38)**	**1.79 (1.39 to 2.30)**
Female versus male	1.04 (0.77 to 1.39)	0.98 (0.58 to 1.66)
Born in Sweden versus born outside Sweden	0.64 (0.41 to 1.00)	0.83 (0.42 to 1.67)
Years of education>12 vs ≤12	0.87 (0.65 to 1.17)	1.13 (0.68 to 1.88)
Comorbidity≥1 versus none	1.03 (0.62 to 1.71)	**2.18 (1.06 to 4.46)**
History of depression†, yes versus no	1.80 (0.77 to 4.21)	1.24 (0.36 to 4.21)
History of anxiety†, yes versus no	1.11 (0.59 to 2.08)	1.05 (0.41 to 2.70)
History of other psychiatric comorbidities†‡, yes versus no	1.10 (0.56 to 2.16)	1.12 (0.41 to 3.07)
History of antidepressants treatment§, yes versus no	0.93 (0.55 to 1.59)	1.79 (0.83 to 3.86)
History of anxiolytics treatment§, yes versus no	1.36 (0.54 to 3.38)	1.06 (0.26 to 4.33)
History of symptomatic fatigue treatment§, yes versus no	1.65 (0.30 to 8.95)	2.13 (0.27 to 16.75)
History of sleeping aids treatment§, yes versus no	0.96 (0.59 to 1.56)	0.71 (0.34 to 1.49)
History of pain treatment§, yes versus no	1.27 (0.93 to 1.75)	**2.36 (1.42 to 3.93)**
DMT		
Rituximab	Ref.	Ref.
Dimethyl fumarate	1.09 (0.79 to 1.51)	0.56 (0.30 to 1.03)
Fingolimod	1.47 (0.80 to 2.73)	2.21 (0.85 to 5.78)
Glatiramer acetate	0.58 (0.29 to 1.14)	0.59 (0.17 to 2.03)
Interferons¶	1.38 (0.98 to 1.93)	1.70 (0.97 to 2.98)
Natalizumab	**0.62 (0.42 to 0.91)**	**0.25 (0.12 to 0.53)**
Teriflunomide	1.44 (0.69 to 2.98)	**4.07 (1.35 to 12.26)**
MS duration>5 years vs ≤5 years	1.07 (0.78 to 1.46)	1.29 (0.80 to 2.08)
Any relapse versus none in the previous year	0.75 (0.55 to 1.03)	**0.49 (0.28 to 0.85)**
Any new cerebral lesions versus none in the previous year	1.21 (0.90 to 1.64)	1.12 (0.69 to 1.80)
SDMT Score	**0.98 (0.97 to 1.00)**	**0.95 (0.92 to 0.97)**
FSMC Score	1.01 (0.99 to 1.04)	1.02 (0.98 to 1.06)
MSIS-29 Physical Score	1.01 (0.99 to 1.03)	1.03 (0.99 to 1.06)
MSIS-29 Psychological Score	1.00 (0.99 to 1.01)	1.00 (0.98 to 1.02)
EQ-5D VAS Score	1.00 (0.98 to 1.01)	1.00 (0.97 to 1.02)
Sick leave previous year**, days	**1.01 (1.00 to 1.01)**	1.00 (1.00 to 1.01)
Disability pension previous year, days**	1.00 (1.00 to 1.01)	1.01 (1.00 to 1.01)

Bold values denote statistical significance at the p < 0.05 level.

*Diagnosed within 5 years prior to DMT start according to the Charlson Comorbidity Index.

†Diagnosed within 5 years prior to DMT start.

‡All mental and behavioural disorders except depression and anxiety disorders.

§Dispensed prescribed drugs within 1 year prior to DMT start.

¶Interferon beta-1a, peginterferon beta 1 a, and interferon beta-1b.

**Restricted to patients 18–64 years old.

DMT, disease-modulating therapy; EDSS, Expanded Disability Status Scale; EQ-VAS, EuroQol Visual Analogue Scale; FSMC, Fatigue Scale for Motor and Cognitive function; MS, multiple sclerosis; MSIS-29, MS Impact Scale; RRMS, relapsing-remitting MS; SDMT, Symbol Digit Modalities Test.

Participants with more than one comorbidity, a history of pain treatment, starting teriflunomide were more likely to belong to the moderate disability trajectory, relative to the no disability one, than participants with no comorbidity, no history of pain treatment, starting rituximab. Participants starting natalizumab, who had a relapse in the year prior to first DMT start, with higher scores on the SDMT at DMT start, were less likely to belong to the minimal disability signs and moderate disability trajectory, relative to the no disability one, than participants starting rituximab, who did not have a relapse in the year prior to first DMT start, with lower scores on the SDTM at DMT start (table 3).

### Membership across processing speed and disability trajectories

**Figure 2:**
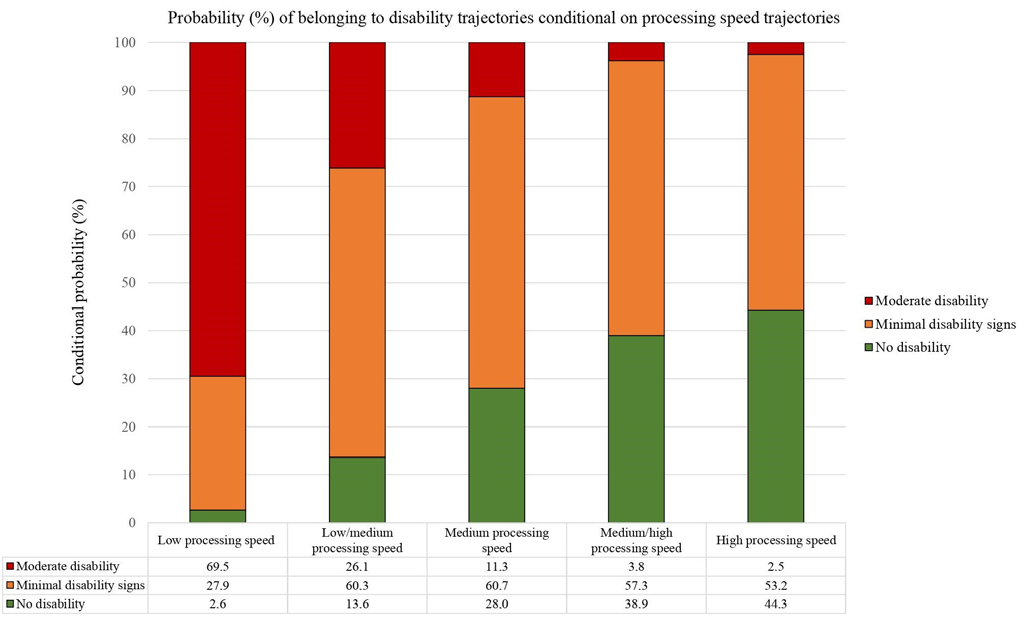
Probabilities of belonging to physical disability trajectories (Expanded Disability Status Scale) conditioned on belonging to each processing speed trajectory (Symbol Digit Modalities Test), quantifying the association between processing speed and physical disability in relapsing-remitting multiple sclerosis patients over 11 years following first disease-modulating therapy initiation.

There was a strong association between cognitive speed and disability trajectories (figure 2). We found that the highest probability (69.5%) of belonging to the moderate physical disability trajectory was among patients belonging to the low processing speed trajectory and that the probability of belonging to the moderate physical disability trajectory decreased for patients belonging to the low/medium (26.1%), medium (11.3%), medium/high (3.8%) and high (2.5%) processing speed trajectory.

### Sensitivity analyses

Excluding participants whose trajectory assignment probability was<0.80 (N=239, 14.5%, for SDMT trajectories and N=102, 6.2 %, for EDSS trajectories) rendered similar predictors of processing speed and disability trajectories (online supplemental eTables 4 and 5).

## Discussion

In this large population-based study investigating processing speed and physical disability trajectories in a contemporary actively treated RRMS cohort, we observe processing speed to remain stable over eleven years from first DMT start. In contrast, patients with a moderate physical disability trajectory deteriorated further over time. While suggesting a higher degree of resilience in cognitive functions, our results nevertheless also show a strong association between lower processing speed and physical disability after DMT start.

Contrary to our expectations, we did not see a deterioration in cognitive speed over time. Thus, although SDMT scores first tended to increase slightly, and later decreased, they never went below baseline values at first DMT start. This is in contrast with the reported worsening of SDMT scores at 5-year and 10-year follow-up.^13 15^ These studies, however, suffer from a 45% dropout rate,^13^ smaller sample sizes of 76^13^ and 680 patients with MS^15^, respectively, and a longer disease duration (9 years) at baseline^13^ (vs 1 year in the present study). It is clear that access to a larger population-based material coupled with the possibility to observe SDMT score changes from first DMT start can explain these differences.^13^ It may also be speculated if results are impacted also by increased used of highly effective DMTs in Sweden compared with other countries.^37^ Nevertheless, we observed that some degree of cognitive difficulties had developed already at first DMT start, as baseline SDMT scores and older age were the strongest predictors of belonging to lower processing speed trajectories, as previously shown.^13 15 21^ Moreover, in line with previous studies,^13 21^ after adjusting for MS severity, we did not see the expected protective effect of cognitive reserve^13^ measured by years of education against decline in SDMT scores in patients with MS,^38^ suggesting that more active MS is associated with cognitive difficulties already at first DMT start.

Our results are in line with a smaller Swedish study showing an improvement in SDMT scores in the 2 years after DMT start, however, solely focusing on natalizumab.^18^ This finding is now extended across all DMTs and further shows that except among patients with low processing speed at DMT start, scores on the SDMT increase up to 5 years after first DMT start and decline thereafter. Although increasing SDMT scores limited to the first years following first DMT start might reflect a test practice effect,^19^ with the specific aim of mitigating this risk, our study participants were routinely administered different SDMT versions. It may be speculated if reduction of inflammatory activity early after start of DMT, plasticity processes and increasing age^39^ can explain these dynamic changes. Further studies are needed to better understand the underlying mechanisms, establish if this improvement is not entirely due to a test practice effect, and if this could be sustained over more than 5 years following first DMT initiation.

Consistent with previous research showing an association between physical disability and processing speed,^12 13 20 23^ this is the first study to show that the link between processing speed and physical disability in patients with MS is not restricted to baseline values but seems to progress in parallel for several years over the course of the disease.

### Strengths and limitations

Major strengths of our study include the large population-based sample size, long follow-up, a rich set of high-quality covariates prospectively collected by healthcare professionals independently linked to national registers. By defining the cohort by first DMT start, we captured a distinct clinical time point relevant to most people with RRMS.

Another strength is having used group-based trajectory modelling to identify processing speed and disability trajectories, which allowed us to account for important between-person variation and heterogeneity of cognitive function and physical disability over time, rather than a single assessment.^32^ However, with the goal of capturing as much population variability as possible, we did not exclude scores on the SDMT collected at short time intervals, potentially contributing to the aforementioned practice effect, and did not add random effects into the model, leading to an increase in the number of groups identified. While this allowed us to identify low and high processing speed groups consisting of a restricted group of patients, we lacked power to detect some of the associations between potential predictors of processing speed trajectories, particularly relevant when investigating the role of first DMTs as predictors of trajectories.

The study also has limitations. Although we addressed missing data by multiple imputation, enabling inclusion of all potential predictors in the analyses, it remains a possibility that residual confounding is affecting the estimated associations with cerebral lesions and MS severity scales. However, our results were in line with complete-case analyses, providing support for not having introduced bias by imputing data. Further, the design of the COMBAT-MS study meant that we included retrospective data prior to 2017, followed by a structured prospective follow-up during 2017–2022. Hence, prior to 2017, overall monitoring, for example, including switch of SDMT versions and visit schedules, was not centrally coordinated, which may impact results, however, likely not comparisons across trajectories. Because we followed participants regardless of DMT switch or discontinuation, which were frequent during this long observation period, we deemed it futile to interpret our results in light of a possible long-term effect of specific DMTs on processing speed trajectories. Finally, despite interpreting our results in light of the conservative interpretation that SDMT is a test of information processing speed, SDMT in individuals with MS can be seen as a valid screening test for cognitive disease impact.^40^

## Conclusions

We observed that RRMS patients’ processing speed remained stable over an average of 7 years following DMT initiation, but that older age and severe disease were associated with cognitive impairment at baseline, in turn highlighting the importance of early diagnosis and intervention to maintain cognitive functions in RRMS. Further research is needed to better understand the underlying mechanisms and identification of high-risk individuals with low processing speed at RRMS diagnosis.

## Data Availability

No data are available.
