# Supplementary material for: Trajectories of cognitive processing speed and physical disability over 11 years following initiation of a first multiple sclerosis disease-modulating therapy
Source: J Neurol Neurosurg Psychiatry. 2023 Aug 9;95(2):134–41. doi: 10.1136/jnnp-2023-331784 (PMC10850621; doi:10.1136/jnnp-2023-331784)
Supplement: Supplementary data [file jnnp-2023-331784supp001.pdf]

Supplementary material

eTable 1: Description and data source of potential predictors of processing speed and disability trajectories included in the analyses

Variable	Description	Data source
**Socio-demographic**		
Age at DMT start	Age at DMT start. Categorized into: 18-34, >34 years.	Total Population Register
Sex	Categorized into: female, male.	Total Population Register
Country of birth	Categorized into: Sweden, outside Sweden.	Total Population Register
Region of residence	Geographical region of residence at DMT start. Categorized into: Northern, Middle, Western South-eastern, Southern Sweden, Stockholm.	MS Registry
Education	Highest education achieved as recorded in the year prior to DMT start. Categorized into: ≤12 years or >12 years of education.	LISA
**Comorbidity**		
CCI	CCI adapted for register-based research in Sweden[1]. It includes myocardial infarction, congestive heart failure, peripheral vascular disease, cerebrovascular disease, pulmonary diseases, rheumatic disease, dementia, hemiplegia, diabetes, chronic kidney disease, liver disease, peptic ulcer disease, cancer and HIV/AIDS. Calculated using comorbidities diagnosed in the 5 years prior to DMT start. Categorized into: mild, (CCI score 1-2), moderate (CCI score 3-4), severe (CCI score ≥5).	Prescribed Drug Register and National Patient Register
History of depression	History of depression recorded in the 5 years prior to DMT start. Defined as a record in the National Patient Register (inpatient and outpatient components, ICD10: F32-F34, F38-F39). Indicator variable (Y/N).	National Patient Register
History of anxiety disorders	History of anxiety disorders recorded in the 5 years prior to DMT start. Defined as a record in the National Patient Register (inpatient and outpatient components, ICD10: F40-F45, F48). Indicator variable (Y/N).	National Patient Register
History of other psychiatric comorbidities	History of other mental or behavioral disorder recorded in the 5 years prior to DMT start. Defined as a record in the National Patient Register (inpatient and outpatient components, F00-F99 except F32-34, F38-39 and F40-48). Indicator variable (Y/N).	National Patient Register
**Treatment dispensation**		
History of antidepressants treatment	History of dispensed antidepressants recorded in the year prior to DMT start. Defined as a record in the Prescribed Drug Register (ATC: N06AB, N06AF, N06AG, N06AX but not N06AX21. N06AA and N06AX21 not included	Prescribed Drug Register

	as commonly used to treat neuropathic pain in MS patients). Indicator variable (Y/N).	
History of anxiolytics treatment	History of dispensed anxiolytics recorded in the year prior to DMT start. Defined as a record in the Prescribed Drug Register (ATC: N05BA12, N05BA06, 05BA06. N05BA01 not included as commonly used to treat other symptoms in MS patients). Indicator variable (Y/N).	Prescribed Drug Register
History of symptomatic treatment for fatigue	History of dispensed central stimulants recorded in the year prior to DMT start. Defined as a record in the Prescribed Drug Register (ATC: N06BA01, N06BA02, N06BA04, N06BA07, N06BA09, N06BA12). Indicator variable (Y/N).	Prescribed Drug Register
History of sleeping aids treatment	History of dispensed sleeping aids recorded in the year prior to DMT start. Defined as a record in the Prescribed Drug Register (ATC: N05CF01, N05CF02, N05CF03, N05CM02, N05CM06, N05CM09, N05CD02, N05CD05). Indicator variable (Y/N).	Prescribed Drug Register
History of pain treatment	History of dispensed pain treatment recorded in the year prior to DMT start. Defined as a record in the Prescribed Drug Register (ATC: N02A, N02BE, M03BB, M03BC, N02BA, N03AX12, N03AX16, N02C, N02BG10, N06AA09, N06AA10). Indicator variable (Y/N).	Prescribed Drug Register
**MS disease-related**		
DMT	Dimethyl fumarate, fingolimod, glatiramer acetate, interferons (interferon beta-1a, peginterferon beta-1a, and interferon beta-1b), natalizumab, rituximab, teriflunomide	MS Registry
MS duration	Number of years from MS diagnosis at DMT start. Categorized into: 0-5, >5.	MS Registry
Relapse	Any relapse within one year prior to DMT start. Indicator variable (Y/N).	MS Registry
New cerebral lesions	Any new cerebral gadolinium-enhancing lesions in the year prior to DMT start. Indicator variable (Y/N).	MS Registry
Processing speed	Processing assessed via SDMT recorded at the most recent visit prior to DMT start. Continuous variable.	MS Registry
Disability	Disability assessed via EDSS recorded at the most recent visit prior to DMT start. Continuous variable.	MS Registry
Fatigue	Fatigue assessed via FSMC total score recorded at the most recent visit prior to DMT start. Continuous variable.	MS Registry
Physical impact of MS	Physical impact of MS assessed via MSIS-29 physical recorded at the most recent visit prior to DMT start. Continuous variable.	MS Registry
Psychological impact of MS	Psychological impact of MS assessed via MSIS-29 physical recorded at the most recent visit prior to DMT start. Continuous variable.	MS Registry
Health-related quality of	Self-rated health assessed via EQ-5D VAS	MS Registry

life	recorded at the most recent visit prior to DMT start. Continuous variable.	
**Productivity loss**		
History of sick leave	Number of sick leave days within one year prior to inclusion DMT start. Continuous variable. Restricted to patients 18-64 years old.	MiDAS
History of disability pension	Number of disability pension days within one year prior to inclusion DMT start. Continuous variable. Restricted to patients 18-64 years old.	MiDAS

Abbreviations: AIDS=acquired immunodeficiency syndrome; ATC= anatomical therapeutic chemical; CCI= Charlson comorbidity index; DMT= disease modulating therapy; EDSS= expanded disability status scale; EQ-5D= european quality of life five dimension; FSMC= fatigue scale for motor and cognitive functions; HIV= human immunodeficiency virus; ICD= international classification of disease; LISA= the longitudinal integrated database for health Insurance and labour market studies; MiDAS= the Swedish social insurance agency; MSIS= multiple sclerosis impact scale; MS= multiple sclerosis; SDMT= symbol digit modalities test; VAS= visual analogue scale.

eTable2: N of number of patients with SDMT and EDSS scores by months from DMT start. Available SDMT and EDSS scores at DMT start reflect the number of available observations prior multiple imputation of baseline values. SDMT and EDSS scores have been incorporated and assigned to the nearest month relative to the DMT start.

Months from DMTstart	SDMT(N)	EDSS(N)
0	874	1,325
1	75	114
2	34	95
3	45	129
4	54	130
5	118	161
6	263	365
7	96	207
8	71	139
9	73	137
10	75	111
11	141	164
12	437	351
13	190	194
14	95	155
15	102	136
16	83	107
17	108	163

18	169	254
19	111	161
20	104	130
21	96	128
22	88	130
23	145	143
24	324	266
25	171	195
26	150	171
27	121	124
28	93	104
29	120	135
30	141	136
31	119	129
32	118	133
33	112	117
34	104	117
35	142	140
36	219	186
37	163	191
38	154	140
39	140	134
40	110	124
41	96	111
42	152	152
43	111	120
44	128	121
45	104	114
46	120	118
47	142	140
48	153	165
49	140	143
50	127	138
51	118	125
52	123	123
53	111	105
54	107	114
55	104	107
56	110	111
57	105	83
58	112	116
59	110	115
60	123	126
61	119	129

62	87	98
63	118	104
64	95	102
65	104	89
66	90	95
67	103	95
68	75	75
69	102	95
70	87	73
71	96	101
72	108	102
73	87	83
74	94	89
75	69	70
76	69	73
77	86	70
78	84	83
79	75	70
80	92	73
81	85	86
82	65	58
83	78	60
84	74	73
85	55	55
86	66	63
87	52	62
88	64	55
89	57	61
90	60	58
91	56	55
92	48	50
93	54	54
94	40	35
95	49	50
96	54	51
97	46	51
98	39	47
99	32	35
100	39	45
101	44	37
102	29	35
103	39	30
104	36	39
105	31	29

106	42	45
107	34	27
108	35	38
109	32	24
110	28	28
111	22	29
112	24	23
113	24	22
114	20	21
115	16	20
116	30	18
117	19	14
118	18	17
119	14	18
120	23	21
121	18	19
122	11	15
123	6	8
124	14	12
125	4	5
126	6	6
127	8	7
128	12	10
129	11	10
130	9	6
131	4	5
132	4	7
133	5	8
134	2	4
Abbreviations: DMT= disease modulating therapy; EDSS= expanded disability status scale; SDMT= symbol digit modalities test.		

eTable 3: Frequency of missing values at DMT start of potential predictors included in the analyses, N=1,645 RRMS patients at first DMT start

	**Missing values at DMT start**
**Covariates**	**N (%)**
Age at DMT start (years)	0 (0.0)
Female	0 (0.0)
Born in Sweden	0 (0.0)
Region of residence	0 (0.0)
Education (years)	4 (0.2)
Comorbidity ≥ 1[a]	0 (0.0)
Depression diagnosis[b]	0 (0.0)
Anxiety diagnosis[b]	0 (0.0)
Other psychiatric comorbidities[b,c]	0 (0.0)
Antidepressants treatment[d]	0 (0.0)
Anxiolytics treatment[d]	0 (0.0)
Symptomatic fatigue treatment[d]	0 (0.0)
Sleeping aids treatment[d]	0 (0.0)
Pain treatment[d]	0 (0.0)
DMT	0 (0.0)
MS duration (years)	5 (0.3)
Relapses	0 (0.0)
New cerebral lesion	186 (11.3)
SDMT score	770 (46.8)
EDSS	319 (19.4)
FSMC total	1372 (83.4)
MSIS-29 physical score	733 (44.6)
MSIS-29 psychological score	733 (44.6)
EQ-5D VAS score	842 (51.2)
Sick leave previous year[e]	0 (0.0)
Disability pension previous year[e]	0 (0.0)

a Diagnosed within five years prior to DMT start according to the CCI.
b Diagnosed within five years prior to DMT start.
c All mental and behavioral disorders except depression and anxiety disorders.
d Dispensed prescribed drugs within one year prior to DMT start.
e Restricted to patients 18-64 years old.

Abbreviations: CCI= Charlson Comorbidity Index; DMT=disease modulating therapy; EDSS=expanded disability status scale; EQ-VAS=euroqol visual analogue scale; FSMC=fatigue scale for motor and cognitive function; MS=multiple sclerosis; MSIS-29=MS impact scale; SDMT= symbol digit modalities test.

eTable 4. ORs of belonging to processing speed trajectories (compared to the low processing speed one) in a multivariable model including region of residence and baseline SDMT in addition to all potential predictors listed, sensitivity analysis of RRMS patients on first DMT whose trajectory assignment probability was ≥ 0.80 (N=1,406)

	**Processing speed trajectories, SDMT starting values**			
	**Low/medium**	**Medium**	**Medium/high**	**High**
	**OR (95% CI)**	**OR (95% CI)**	**OR (95% CI)**	**OR (95% CI)**
**Age, years**				
18-34	Ref.	Ref.	Ref.	Ref.
>34	0.40 (0.13; 1.22)	0.26 (0.08; 0.87)	**0.16 (0.05; 0.56)**	**0.11 (0.03; 0.39)**
**Female vs. male**	2.25 (0.38; 13.16)	4.05 (0.69; 23.92)	4.36 (0.69; 27.68)	2.75 (0.37; 20.32)
**Born in Sweden vs. born outside Sweden**	0.24 (0.02; 3.28)	0.32 (0.02; 4.84)	0.36 (0.02; 5.80)	0.24 (0.01; 5.16)
**Years of education >12 vs ≤12**	1.26 (0.22; 7.16)	1.50 (0.25; 9.06)	1.20 (0.18; 8.15)	1.57 (0.19; 12.80)
**Comorbidity ≥ 1[a] vs. none**	0.37 (0.03; 5.26)	0.21 (0.01; 3.32)	0.09 (0.00; 1.76)	0.05 (0.00; 1.29)
**History of depression[b], yes vs. no**	0.18 (0.00; 15.11)	0.16 (0.00; 14.31)	0.07 (0.00; 10.20)	n/a
**History of anxiety[b], yes vs. no**	7.50 (0.06; 1011.56)	7.75 (0.06; 1066.70)	4.04 (0.03; 602.58)	n/a
**History of other psychiatric comorbidities[b,c], yes vs. no**	0.13 (0.00; 4.26)	0.06 (0.00; 2.30)	0.03 (0.00; 1.64)	n/a
**History of antidepressants treatment[d], yes vs. no**	0.93 (0.07; 12.53)	0.71 (0.05; 10.85)	0.79 (0.04; 16.12)	1.13 (0.04; 33.22)
**History of anxiolytics treatment[d], yes vs.no**	0.38 (0.01; 13.63)	0.49 (0.01; 20.02)	0.90 (0.02; 53.81)	n/a
**History of symptomatic fatigue treatment[d], yes vs. no**	n/a	n/a	n/a	n/a
**History of sleeping aids treatment[d], yes vs. no**	2.55 (0.18; 37.03)	2.72 (0.17; 43.59)	2.16 (0.12; 40.42)	5.33 (0.24; 116.32)
**History of pain treatment[d], yes vs. no**	0.68 (0.11; 4.17)	0.62 (0.09; 4.17)	0.55 (0.07; 4.17)	0.26 (0.03; 2.22)
**DMT**				
Rituximab	Ref.	Ref.	Ref.	Ref.
Dimethyl fumarate	n/a	n/a	n/a	n/a
Fingolimod	n/a	n/a	n/a	n/a
Glatiramer acetate	n/a	n/a	n/a	n/a
Interferons[e]	n/a	n/a	n/a	n/a
Natalizumab	n/a	n/a	n/a	n/a
Teriflunomide	n/a	n/a	n/a	n/a
**MS duration >5 years vs. ≤5 years**	1.05 (0.14; 7.84)	0.99 (0.12; 8.07)	0.71 (0.08; 6.10)	n/a
**Any relapse vs. none in the previous year**	4.13 (0.50; 33.93)	4.95 (0.54; 45.37)	5.54 (0.55; 55.96)	5.49 (0.51; 59.45)
**Any new cerebral lesions vs. none in the previous year**	0.23 (0.03; 2.13)	0.33 (0.03; 3.19)	0.22 (0.02; 2.42)	0.23 (0.02; 2.97)
**EDSS score**	0.81 (0.38; 1.72)	0.73 (0.33; 1.60)	0.72 (0.31; 1.66)	0.77 (0.30; 2.02)
**FSMC score**	1.08 (0.97; 1.20)	1.07 (0.95; 1.20)	1.07 (0.95; 1.21)	1.06 (0.91; 1.24)
**MSIS-29 physical score**	0.94 (0.86; 1.03)	0.92 (0.84; 1.02)	0.93 (0.83; 1.03)	0.94 (0.83; 1.06)
**MSIS-29 psychological score**	1.00 (0.93; 1.07)	1.01 (0.93; 1.09)	1.00 (0.92; 1.08)	0.99 (0.90; 1.08)
**EQ-5D VAS score**	1.01 (0.95; 1.07)	1.01 (0.95; 1.07)	1.02 (0.94; 1.09)	1.02 (0.94; 1.10)
**Sick leave previous year[f], days**	0.99 (0.97; 1.01)	0.99 (0.97; 1.01)	0.99 (0.97; 1.01)	0.99 (0.97; 1.01)
**Disability pension previous year, days[f]**	1.00 (0.99; 1.01)	1.00 (0.98; 1.01)	1.00 (0.98; 1.02)	0.92 (0.03; 24.10)

a Diagnosed within five years prior to DMT start according to the CCI.

b Diagnosed within five years prior to DMT start.
c All mental and behavioral disorders except depression and anxiety disorders.
d Dispensed prescribed drugs within one year prior to DMT start.
e Interferon beta-1a, peginterferon beta-1a, and interferon beta-1-b.
f Restricted to patients 18-64 years old.

Abbreviations: CCI= Charlson comorbidity index; CI=confidence interval; DMT=disease modulating therapy; EDSS=expanded disability status scale; EQ-VAS=euroqol visual analogue scale; FSMC=fatigue scale for motor and cognitive function; MS=multiple sclerosis; MSIS-29=MS impact scale; n/a=not applicable; OR=odds ratio; RRMS= relapsing-remitting MS; SDMT= symbol digit modalities test.

eTable 5. ORs of belonging to disability trajectories (compared to the no disability one) in a multivariable model including region of residence and baseline EDSS in addition to all potential predictors listed, sensitivity analysis of RRMS patients on first DMT whose trajectory assignment probability was ≥ 0.80 (N=1,543)

	**Disability trajectories, EDSS starting values**	
	**Minimal disability signs**	**Moderate disability**
	**OR (95% CI)**	**OR (95% CI)**
**Age, years**		
18-34	Ref.	Ref.
>34	1.14 (0.97; 1.35)	**1.77 (1.29; 2.42)**
**Female vs. male**	1.07 (0.76; 1.51)	0.91 (0.50; 1.67)
**Born in Sweden vs. born outside Sweden**	**0.50 (0.30; 0.83)**	0.84 (0.37; 1.93)
**Years of education >12 vs ≤12**	0.90 (0.65; 1.24)	1.16 (0.65; 2.06)
**Comorbidity ≥ 1[a] vs. none**	1.15 (0.67; 1.98)	**3.35 (1.47; 7.60)**
**History of depression[b], yes vs. no**	1.78 (0.66; 4.78)	2.04 (0.45; 9.29)
**History of anxiety[b], yes vs. no**	0.91 (0.44; 1.91)	0.58 (0.18; 1.85)
**History of other psychiatric comorbidities[b,c], yes vs. no**	1.81 (0.77; 4.29)	1.30 (0.34; 4.89)
**History of antidepressants treatment[d], yes vs. no**	0.93 (0.50; 1.73)	1.89 (0.74; 4.83)
**History of anxiolytics treatment[d], yes vs.no**	1.75 (0.62; 4.89)	1.20 (0.26; 5.63)
**History of symptomatic fatigue treatment[d], yes vs. no**	n/a	n/a
**History of sleeping aids treatment[d], yes vs. no**	0.92 (0.53; 1.59)	0.82 (0.34; 1.94)
**History of pain treatment[d], yes vs. no**	1.45 (1.01; 2.08)	**2.55 (1.40; 4.66)**
**DMT**		
Rituximab	Ref.	Ref.
Dimethyl fumarate	1.17 (0.81; 1.69)	0.63 (0.30; 1.31)
Fingolimod	1.22 (0.63; 2.39)	1.83 (0.62; 5.36)
Glatiramer acetate	0.61 (0.28; 1.30)	0.81 (0.17; 3.75)
Interferons[e]	**1.51 (1.06; 2.17)**	1.65 (0.88; 3.09)
Natalizumab	**0.52 (0.34; 0.79)**	**0.15 (0.07; 0.35)**
Teriflunomide	1.26 (0.55; 2.88)	**4.53 (1.18; 17.48)**
**MS duration >5 years vs. ≤5 years**	1.01 (0.71; 1.45)	1.21 (0.69; 2.13)
**Any relapse vs. none in the previous year**	0.70 (0.49; 1.00)	**0.48 (0.26; 0.90)**
**Any new cerebral lesions vs. none in the previous year**	1.37 (0.99; 1.89)	1.29 (0.72; 2.33)
**SDMT score**	**0.98 (0.96; 0.99)**	**0.93 (0.91; 0.96)**
**FSMC score**	1.01 (0.98; 1.03)	1.00 (0.96; 1.05)
**MSIS-29 physical score**	1.02 (0.99; 1.05)	1.04 (1.00; 1.09)
**MSIS-29 psychological score**	0.99 (0.98; 1.01)	0.99 (0.97; 1.01)

**EQ-5D VAS score**	1.00 (0.98; 1.01)	0.99 (0.97; 1.02)
**Sick leave previous year[f], days**	**1.01 (1.00; 1.01)**	1.00 (1.00; 1.01)
**Disability pension previous year, days[f]**	1.00 (1.00; 1.01)	1.00 (1.00; 1.01)

a Diagnosed within five years prior to DMT start according to the CCI.
b Diagnosed within five years prior to DMT start.
c All mental and behavioral disorders except depression and anxiety disorders.
d Dispensed prescribed drugs within one year prior to DMT start.
e Interferon beta-1a, peginterferon beta-1a, and interferon beta-1b.
f Restricted to patients 18-64 years old.

Abbreviations: CCI= Charlson comorbidity index; CI=confidence interval; DMT=disease modulating therapy; EDSS=expanded disability status scale; EQ-VAS=euroqol visual analogue scale; FSMC=fatigue scale for motor and cognitive function; MS=multiple sclerosis; MSIS-29=MS impact scale; OR=odds ratio; RRMS= relapsing-remitting MS; SDMT= symbol digit modalities test.

References for supplementary material

1. Ludvigsson JF, Appelros P, Askling J, et al. Adaptation of the Charlson Comorbidity Index for Register-Based Research in Sweden. *Clinical epidemiology.* 2021;13:21-41.
